# A Comparative Transcriptomic Analysis Reveals That HSP90AB1 Is Involved in the Immune and Inflammatory Responses to Porcine Deltacoronavirus Infection

**DOI:** 10.3390/ijms23063280

**Published:** 2022-03-18

**Authors:** Yujia Zhao, Rui Chen, Dai Xiao, Luwen Zhang, Daili Song, Yiping Wen, Rui Wu, Qin Zhao, Senyan Du, Xintian Wen, Sanjie Cao, Xiaobo Huang

**Affiliations:** 1 Research Center for Swine Diseases, College of Veterinary Medicine, Sichuan Agricultural University, Chengdu 611130, China; b20172403@stu.sicau.edu.cn (Y.Z.); chenrui@stu.sicau.edu.cn (R.C.); xiaodai@stu.sicau.edu.cn (D.X.); zhangluwen@stu.sicau.edu.cn (L.Z.); songdaili@stu.sicau.edu.cn (D.S.); wyp@sicau.edu.cn (Y.W.); wurui1977@sicau.edu.cn (R.W.); zhao.qin@sicau.edu.cn (Q.Z.); senyandu@sicau.edu.cn (S.D.); xintian@sicau.edu.cn (X.W.); csanjie@sicau.edu.cn (S.C.); 2 Sichuan Science-Observation Experimental Station for Veterinary Drugs and Veterinary Diagnostic Technology, Ministry of Agriculture, Chengdu 611130, China; 3 National Animal Experiments Teaching Demonstration Center, Sichuan Agricultural University, Chengdu 611130, China

**Keywords:** PDCoV, HSP90AB1, transcriptomic analysis, immune and inflammatory response

## Abstract

PDCoV is an emerging enteropathogenic coronavirus that mainly causes acute diarrhea in piglets, seriously affecting pig breeding industries worldwide. To date, the molecular mechanisms of PDCoV-induced immune and inflammatory responses or host responses in LLC-PK cells in vitro are not well understood. HSP90 plays important roles in various viral infections. In this study, HSP90AB1 knockout cells (HSP90AB1^KO^) were constructed and a comparative transcriptomic analysis between PDCoV-infected HSP90AB1^WT^ and HSP90AB1^KO^ cells was conducted using RNA sequencing to explore the effect of HSP90AB1 on PDCoV infection. A total of 1295 and 3746 differentially expressed genes (DEGs) were identified in PDCoV-infected HSP90AB1^WT^ and HSP90AB1^KO^ cells, respectively. Moreover, most of the significantly enriched pathways were related to immune and inflammatory response-associated pathways upon PDCoV infection. The DEGs enriched in NF-κB pathways were specifically detected in HSP90AB1^WT^ cells, and NF-κB inhibitors JSH-23, SC75741 and QNZ treatment reduced PDCoV infection. Further research revealed most cytokines associated with immune and inflammatory responses were upregulated during PDCoV infection. Knockout of HSP90AB1 altered the upregulated levels of some cytokines. Taken together, our findings provide new insights into the host response to PDCoV infection from the transcriptome perspective, which will contribute to illustrating the molecular basis of the interaction between PDCoV and HSP90AB1.

## 1. Introduction

Porcine deltacoronavirus (PDCoV), a member of the emerging genus deltacoronavirus, in the family coronaviridae causes enteric disease in piglets characterized by diarrhea, vomiting and dehydration [1]. Since its first outbreak in the United States in 2014, PDCoV infection has gradually been reported in many pig-producing countries worldwide, resulting in substantial economic losses for the swine industry [2,3]. Similar to porcine epidemic diarrhea virus (PEDV) and transmissible gastroenteritis virus (TGEV), PDCoV is an enveloped, single-stranded, positive-sense RNA virus with a genome of approximately 25.4 kb in length that encodes 15 mature nonstructural proteins (NSPs), four structural proteins, including spike protein (S), membrane protein (M), nucleocapsid protein (N) and envelope protein (E), and three accessory proteins (NS6, NS7 and NS7a) [4,5]. To date, numerous studies have confirmed that PDCoV proteins play important roles in the recognition of receptors [6], virus entry [7], assembly [8] replication [9], immune regulation [10], and other processes. However, the molecular mechanisms of PDCoV-induced immune and inflammatory responses or host responses in LLC-PK cells in vitro are not well understood.

Viral infection activates the host innate immune response, which is the first line of defense against viral invasion. Recent reports have identified that PDCoV infection of host cells, including porcine kidney cells (PK15 and LLC-PK) [11,12], swine testicular (ST) cells [13] and porcine small intestinal epithelial cells (IPEC-J2) [14,15], stimulates innate immune response-associated pathways in response to viral infection. In addition, further research indicated that human intestinal epithelial cells (HIECs) exhibit a more pronounced response to PDCoV infection than IPEC-J2 cells [14]. Nevertheless, viruses have also evolved diverse strategies to antagonize host antiviral immune responses by inhibiting interferon (IFN) production or disrupting IFN-associated signaling pathways and successfully completing replication in host cells. For example, PDCoV infection reduces the number of peroxisomes and inhibits interferon regulatory factor 1 (IRF1) nuclear translocation to further antagonize the production of IFN-λ1 [16]. PDCoV NSP15 inhibits IFN-β production independently of its endoribonuclease activity by impairing nuclear factor-κB (NF-κB) activation [17]. Moreover, the PDCoV N and NS6 proteins directly interact with retinoic acid-inducible gene I (RIG-I) to interfere with the binding of RIG-I to double-stranded RNA (dsRNA), thereby inhibiting IFN-β production [18,19]. The other PDCoV nonstructural proteins, NSP5 [20] and NSP10 [21], as well as the accessory protein NS7a [22], were also identified as innate immunity antagonists. Autophagy is also a defense mechanism against viral infection [23,24]. However, several studies have shown that viruses utilize the autophagy pathway of host cells to promote replication [25,26]. PDCoV infection has been reported to induce autophagy [27]. Afterward, Duan et al. [28] showed that PDCoV activated autophagy via the p38 signaling pathway, further promoting its replication in vitro and in vivo.

Heat shock protein 90 (HSP90) is a family of highly conserved molecular chaperones that play crucial roles in the folding, maturation and activation of their client proteins to maintain cellular homeostasis and survival [29]. In addition to protein kinases and transcription factors, numerous viral proteins are HSP90 client proteins [30,31,32]. Moreover, HSP90 facilitates viral infection by stabilizing viral proteins, preventing their degradation by the ubiquitin–proteasome pathway [33] or autophagy-mediated degradation pathway [34]. The interaction of HSP90 with its client proteins also regulates viral infection by modulating various cellular signaling pathways. For example, the binding of HSP90 to flavivirus NS5 protein prevents its interaction with client kinases, resulting in the suppression of JAK/STAT-dependent cytokine signaling [35]. HSP90AB1 interacts with stimulator of interferon genes (STING) to stabilize its protein level, leading to the activation of the downstream target TANK-binding kinase 1 (TBK1) to induce IFN production in response to herpes simplex virus 1 (HSV-1) infection [36]. Hepatitis B virus polymerase inhibits IκB kinase (IKK) activity by interacting with HSP90AB1, further suppressing NF-κB-mediated gene transcription [37]. Recently, Li et al. [33] revealed that HSP90 is a critical host factor associated with human coronavirus middle east respiratory syndrome coronavirus (MERS-CoV), severe acute respiratory syndrome coronavirus 2 (SARS-CoV-2) and SARS-CoV. However, no reports have described the role and function of HSP90 in porcine coronavirus infection.

In our previous study, HSP90AB1 was identified as a host factor that could potentially interact with PDCoV by co-immunoprecipitation (Co-IP) coupled with LC/MS-MS [38]. Moreover, we also found that HSP90 inhibitors 17-AAG that targets both HSP90AA1 and HSP90AB1, and VER-82576 that targets only HSP90AB1 could reduce PDCoV infection. However, KW-2478, which binds HSP90AA1 with high affinity had no significant inhibitory effect on PDCoV infection [39]. In this study, we aimed to identify the effect of HSP90AB1 knockout on PDCoV infection and further analyze the transcriptomic alterations in PDCoV-infected HSP90AB1^WT^ and HSP90AB1^KO^ cells. Our results indicated that HSP90AB1 exerted significant effects on PDCoV infection. Moreover, the identified differentially expressed genes (DEGs) were significantly enriched in immune and inflammatory response-associated signaling pathways. To our knowledge, this report is the first to investigate the role of HSP90AB1 in PDCoV infection from the perspective of a transcriptome analysis.

## 2. Results

### 2.1. HSP90AB1 Knockout Reduces PDCoV Infection

**Figure 1:**
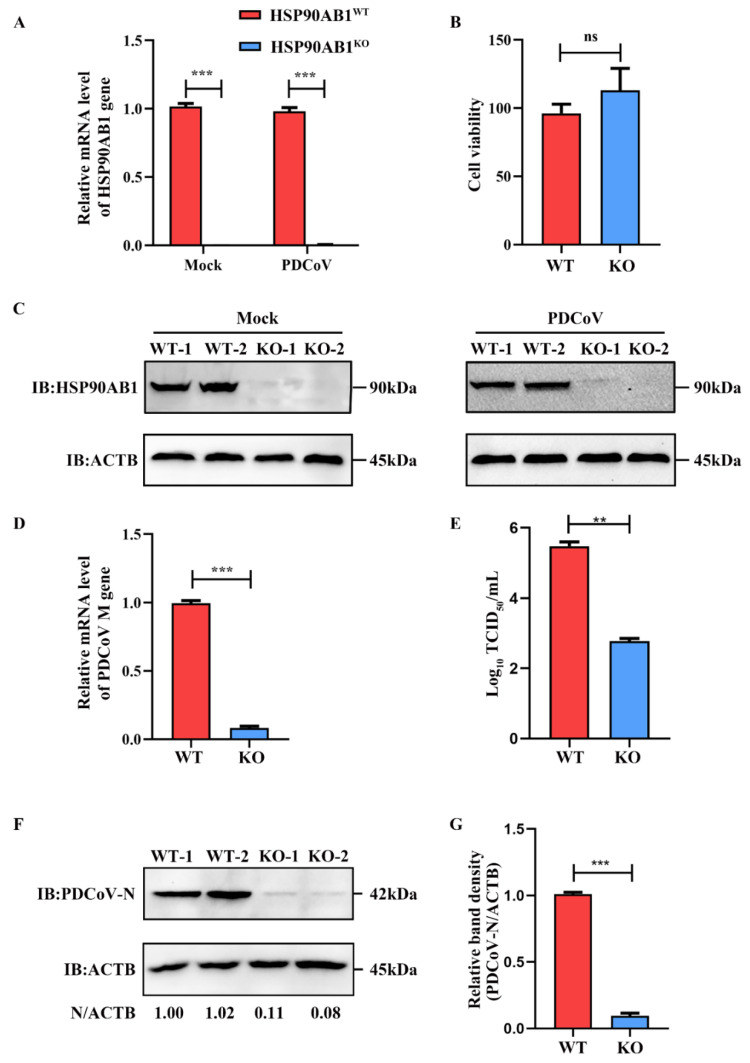
HSP90AB1 knockout reduces PDCoV infection. (**A**) The mRNA level of HSP90AB1 in HSP90AB1^WT^ and HSP90AB1^KO^ cells was detected using qRT-PCR. (**B**) CCK-8 assay of the viability of HSP90AB1^WT^ and HSP90AB1^KO^ cells. (**C**) The HSP90AB1 protein expression level in HSP90AB1^WT^ and HSP90AB1^KO^ cells was analyzed using western blotting. ACTB was used as the sample loading control. (**D**) Detection of the mRNA level of the PDCoV *M* gene using qRT-PCR. (**E**) The virus titer in the culture supernatants was determined using TCID_50_ assay. (**F**) Detection of the PDCoV N protein level using western blotting. ACTB was used as the sample loading control. (**G**) The band density of the protein was quantified using ImageJ software, and the PDCoV-N to ACTB ratios were normalized to the control. ** means *p* ≤ 0.01, *** means *p* ≤ 0.001.

The CRISPR/Cas9 system was used to construct HSP90AB1 knockout cell line as a method to evaluate the role of HSP90AB1 in PDCoV infection. qRT-PCR and western blotting results indicated that HSP90AB1 mRNA level and protein expression level was rarely detected in the mock- and PDCoV-infected cells (Figure 1A,C). Moreover, CCK-8 results showed that HSP90AB1 knockout had no detectable effect on cell viability (Figure 1B). HSP90AB1^KO^ cells were then used to assess the contribution of HSP90AB1 to PDCoV infection. As shown in Figure 1D, HSP90AB1 knockout reduced the mRNA level of PDCoV *M* gene by approximately 92% compared with HSP90AB1^WT^ cells, as determined using qRT-PCR. The virus titer in the culture supernatants was reduced by approximately 2.7 log_10_ TCID_50_/mL (Figure 1E). Western blotting analysis showed a 90% reduction in the level of PDCoV N protein compared with that in HSP90AB1^WT^ cells (Figure 1F,G). Taken together, knockout of endogenous HSP90AB1 expression in LLC-PK cells reduced PDCoV infection, suggesting that the HSP90AB1 protein is a critical factor for PDCoV infection.

**Figure 2:**
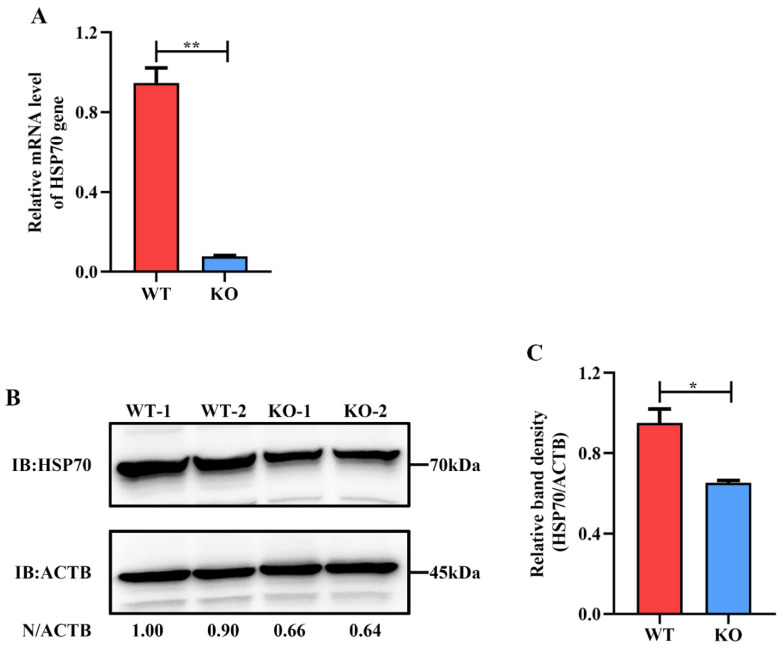
HSP90AB1 knockout reduces HSP70 expression level. (**A**) The mRNA level of HSP70 in HSP90AB1^WT^ and HSP90AB1^KO^ cells was detected using qRT-PCR. (**B**) Detection of the HSP70 protein level using western blotting. ACTB was used as the sample loading control. (**C**) The band density of the protein was quantified using ImageJ software, and the HSP70 to ACTB ratios were normalized to the control. * means *p* ≤ 0.05, ** means *p* ≤ 0.01.

Moreover, the expression of HSP70 in HSP90AB1^WT^ and HSP90AB1^KO^ cells was also detected by qRT-PCR and western blotting. Results indicated that knockout of HSP90AB1 led to a 90% reduction of *HSP70* mRNA level and a 40% decrease of HSP70 protein expression level (Figure 2A–C), suggesting that HSP70 may serve as a cochaperone for HSP90AB1.

### 2.2. Quality Control and Mapping of RNA Sequencing Results

Eight transcriptome libraries were constructed from mock- and PDCoV-infected cell samples to explore the alteration in the transcriptome profile between HSP90AB1^WT^ and HSP90AB1^KO^ cells during PDCoV infection. Through RNA sequencing, approximately 585.0 million raw reads were initially generated, with approximately 73.1 million reads per sample. After quality control, approximately 583.6 million clean reads remained, with approximately 10.9 G bases per sample, and the Q20 and Q30 percentages of clean data for all samples were more than 97% and 92%, respectively. The GC content of clean reads in each sample ranged from 48.03% to 48.88% (Table 1). These findings indicated that the resulting clean reads were of high quality and suitable for further analysis.

**Figure 3:**
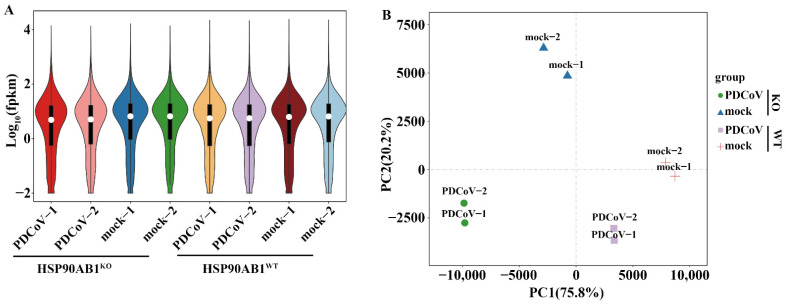
Differentially expressed genes (DEGs) in HSP90AB1^WT^ and HSP90AB1^KO^ cells were analyzed following mock and PDCoV infection. (**A**) Violin plot of gene expression patterns for each sample, with the origin representing the median. (**B**) Principal component analysis (PCA) of eight samples of mock- or PDCoV-infected HSP90AB1^WT^ or HSP90AB1^KO^ cells.

For further analysis, the high-quality clean reads were subsequently mapped to the reference genome after removing the rRNA-mapped reads. As shown in Table 2, more than 85.2% of the clean reads was successfully mapped to the reference genome, and the unique mapped reads of eight samples ranged from 82.8% to 92.9%. In addition, more than 76.03% of the mapped sequences was located in the exonic region, while the percentages of intronic and intergenic sequences was ranged from 17.16% and 2.82% to 20.96% and 3.51%, respectively. Simultaneously, 17,557 genes, including 604 novel genes, were also identified as expressed genes after the quantification of gene expression levels as FPKM (Table 2 and Table S1), and the distribution of gene expression in each sample is shown in Figure 3A.

Subsequently, Pearson’s correlation analysis and Principal component analysis (PCA) were carried out based on the expression level in each sample. As shown in Figure 3B, mock-infected HSP90AB1^WT^ or HSP90AB1^KO^ cells did not cluster with the cells infected with PDCoV. Similarly, the two different cell lines were also clearly distinct from each other. Moreover, the correlation coefficients between two independent biological replicates exceeded 0.991, and the mock- and PDCoV-infected HSP90AB1^WT^ or HSP90AB1^KO^ cells exhibited weak correlations with each other (Figure S1). Based on these results, the samples within a group had better repeatability and similar samples clustered together.

### 2.3. Identification and Analysis of DEGs

**Figure 4:**
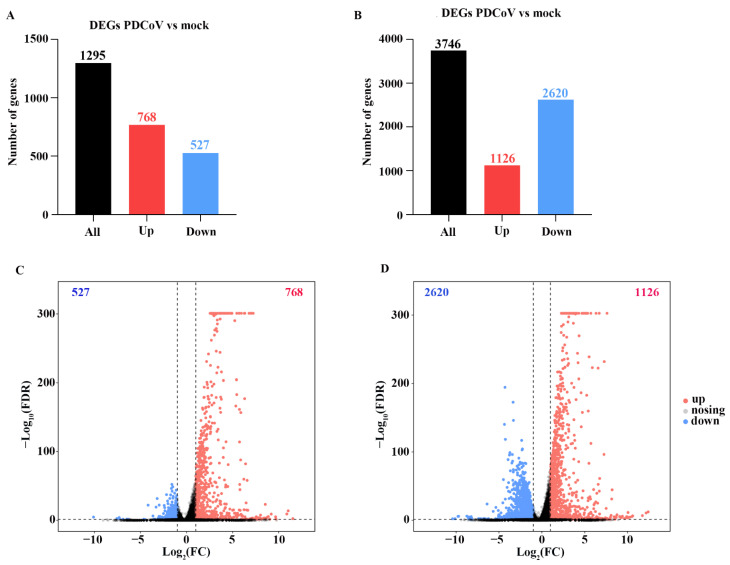
DEGs in HSP90AB1^WT^ and HSP90AB1^KO^ cells were analyzed following mock and PDCoV infection. (**A**,**B**) The numbers of upregulated and downregulated DEGs in HSP90AB1^WT^ (**A**) and HSP90AB1^KO^ (**B**) cells were evaluated. (**C**,**D**) Volcano plot showing the DEGs in HSP90AB1^WT^ (**C**) and HSP90AB1^KO^ (**D**) cells. The upregulated DEGs are represented by red dots, downregulated DEGs are represented by blue dots, and genes with no significant differences in expression are represented by gray dots.

The expression of identified genes from HSP90AB1^WT^ and HSP90AB1^KO^ cells was screened by comparing mock- and PDCoV-infected cells, respectively, under the condition of a false discovery rate (FDR) ≤ 0.05 and |log_2_ fold change| ≥ 1, and the identified DEGs are listed in Tables S2 and S3. Notably, 1295 DEGs were identified in PDCoV-infected HSP90AB1^WT^ cells, of which 768 genes were upregulated and 527 genes were downregulated (Figure 4A,C). However, PDCoV-infected HSP90AB1^KO^ cells exhibited the differential expression of 3746 genes, of which 1126 genes were upregulated and 2620 were downregulated (Figure 4B,D). Thus, more DEGs were identified in HSP90AB1^KO^ cells than in HSP90AB1^WT^ cells upon PDCoV infection.

### 2.4. GO Enrichment Analysis of the DEGs upon PDCoV Infection

**Figure 5:**
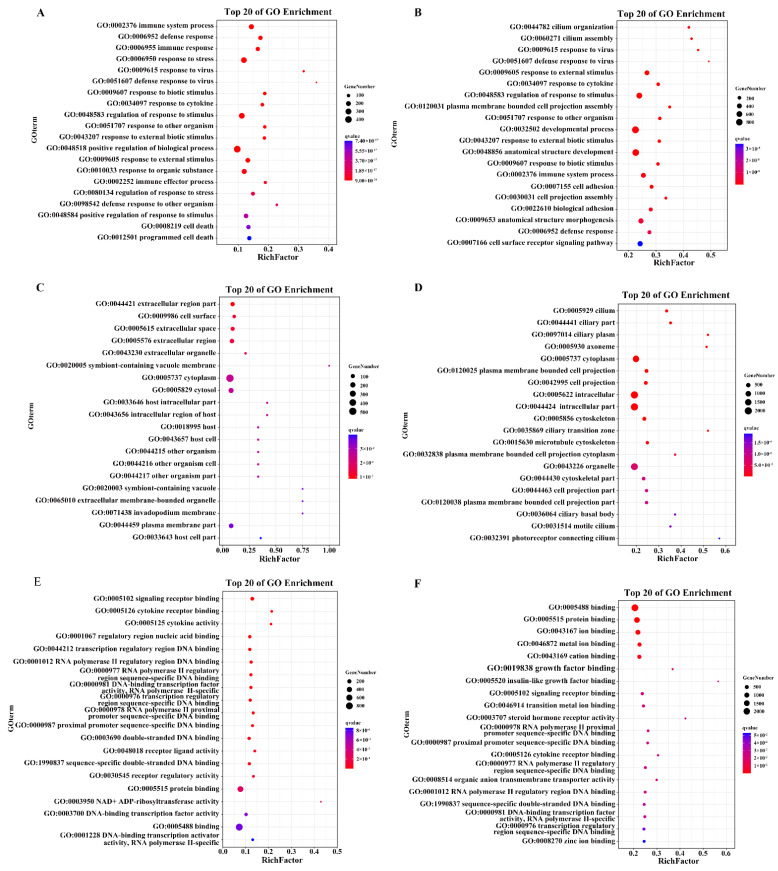
Bubble map of the top 20 most enriched GO terms. (**A**,**B**) The top 20 significantly enriched GO terms in HSP90AB1^WT^ (**A**) and HSP90AB1^KO^ (**B**) cells in the BP category. (**C**,**D**) The top 20 significantly enriched GO terms in HSP90AB1^WT^ (**C**) and HSP90AB1^KO^ (**D**) cells in the CC category. (**E**,**F**) The top 20 significantly enriched GO terms in HSP90AB1^WT^ (**E**) and HSP90AB1^KO^ (**F**) cells in the MF category. Enriched terms were selected with a corrected *p*-value ≤ 0.05. The dot size indicates the number of enriched DEGs, and the dot color indicates the Q-value.

GO enrichment analysis was performed to categorize and annotate DEGs into three categories, biological processes (BP), cellular components (CC), and molecular functions (MF), and to determine the functions of the DEGs and possible biological processes involved in HSP90AB1^WT^ and HSP90AB1^KO^ cells. The identified DEGs in HSP90AB1^WT^ and HSP90AB1^KO^ cells were categorized into 54 and 56 different GO terms, respectively, and the number of upregulated or downregulated DEGs in HSP90AB1^KO^ cells that enriched in each GO term was higher than the number in HSP90AB1^WT^ cells (Figure S2). In the BP category, 820 and 378 significant GO terms were enriched in HSP90AB1^WT^ and HSP90AB1^KO^ cells, respectively. Among the top 20 enriched GO terms in HSP90AB1^WT^ cells, most DEGs were enriched in processes related to the positive regulation of biological processes, regulation of response to stimulus, response to stress and immune system process (Figure 5A). In HSP90AB1^KO^ cells, development processes, regulation of response to stimulus, response to external stimulus, immune system process and cell surface receptor signaling pathways showed the highest enrichment, except for anatomical structure development and anatomical structure morphogenesis (Figure 5B). Moreover, the analysis of enriched CC and MF terms revealed that the most highly annotated GO terms were cell and cell parts, binding and protein binding, respectively (Figure 5C–E). The details of the GO terms are shown in Tables S4 and S5.

### 2.5. KEGG Pathway Analysis of the DEGs upon PDCoV Infection

**Figure 6:**
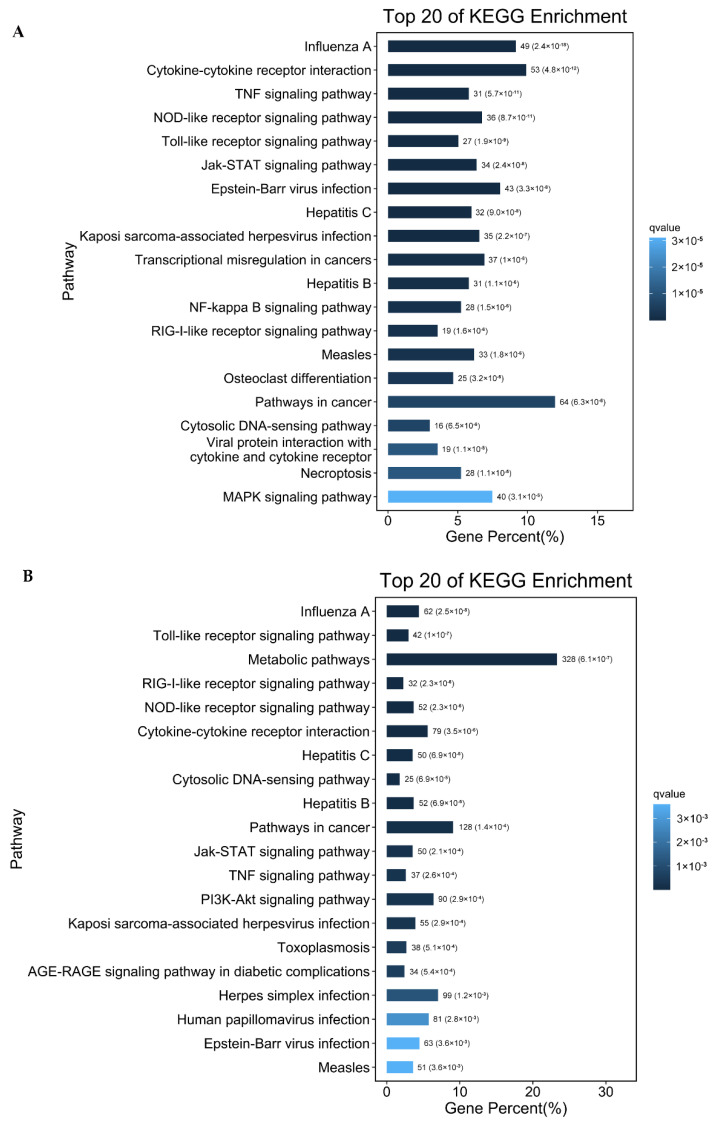
Histograms of the top 20 most enriched signaling pathways. (**A**) The top 20 significantly enriched pathways were identified in HSP90AB1^WT^ cells. (**B**) The top 20 significantly enriched pathways identified in HSP90AB1^KO^ cells. Enriched pathways were selected with a corrected *p*-value ≤ 0.05. The length indicates the percentage of genes, and the color indicates the Q-value.

The DEGs in HSP90AB1^WT^ and HSP90AB1^KO^ cells were submitted to the KEGG database to further identify the possible signaling pathways existing among DEGs following PDCoV infection. As shown in Figure S3, most of the identified DEGs in both cell lines were mainly related to the categories of signal transduction, immune system, infectious disease and cancer, with the only exception of global and overview maps in HSP90AB1^KO^ cells, and a greater number of DEGs being enriched in signal transduction pathways than in other pathways. Moreover, further analysis indicated that 52 and 45 signaling pathways were significantly enriched in HSP90AB1^WT^ and HSP90AB1^KO^ cells upon PDCoV infection, respectively (Tables S6 and S7). Among the top 20 relevant signaling pathways, the RIG-I-like receptor signaling pathway, NOD-like receptor signaling pathway, TNF signaling pathway, Toll-like receptor signaling pathway, Jak-STAT signaling pathway, cytosolic DNA-sensing pathway and cytokine-cytokine receptor interaction pathway were enriched in both cell lines and were related to immune and inflammatory responses (Figure 6). Interestingly, the NF-κB signaling pathway, MAPK signaling pathway and viral protein interaction with cytokine and cytokine receptor were specifically identified in HSP90AB1^WT^ cells, and the NF-κB signaling pathway identified in HSP90AB1^KO^ cells was not significantly enriched pathway upon PDCoV infection (Figure 6A,B). Moreover, the PI3K-AKT signaling pathway was specifically enriched in HSP90AB1^KO^ cells (Figure 6B).

### 2.6. Expression of DEGs in Immune- and Inflammatory-Related Pathways

**Figure 7:**
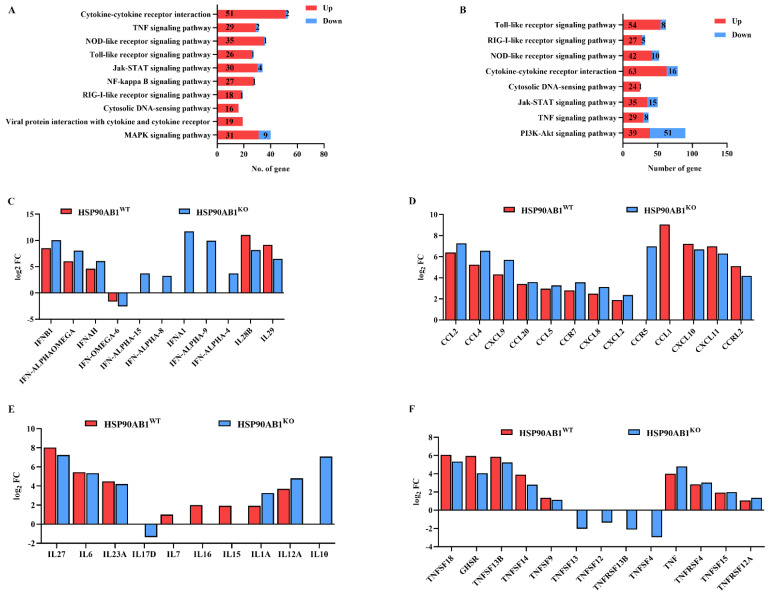
Immune and inflammatory responses in PDCoV-infected HSP90AB1^WT^ and HSP90AB1^KO^ cells. (**A,B**) KEGG analysis of immune- and inflammation-related signaling pathways in HSP90AB1^WT^ (**A**) and HSP90AB1^KO^ (**B**) cells following PDCoV infection. (**C**) The interferon-related genes. (**D**) The chemokine and chemokine receptor related genes. (**E**) The interleukin-related genes. (**F**) Tumor necrosis factor-related genes.

The expression of DEGs related to immune and inflammatory responses in HSP90AB1^WT^ and HSP90AB1^KO^ cells was further analyzed. KEGG enrichment results showed that most of the DEGs related to immune and inflammatory responses were considerably upregulated in HSP90AB1^WT^ and HSP90AB1^KO^ cells (Figure 7A,B). Upon PDCoV infection, the expression of IFNs increased significantly with the exception of IFN-OMGEA-6, among which IFNA1 was the most substantially upregulated IFN, increasing by approximately 2000-fold in HSP90AB1^KO^ cells (Figure 7C). The expression levels of chemokines and chemokine receptors increased substantially following PDCoV infection, among which the most increased were CCL2, CCL4, CXCL11 and CXCL10 in both cells (Figure 7D). Several interleukin (IL) members, such as IL27 and IL6, TNF superfamily (TNFSF) members and TNF receptor superfamily (TNFRS) members, including TNFSF18, GHSR and TNFSF13B, were also significantly upregulated (Figure 7E,F).

### 2.7. Validation of Transcriptomic Data Using qRT-PCR

**Figure 8:**
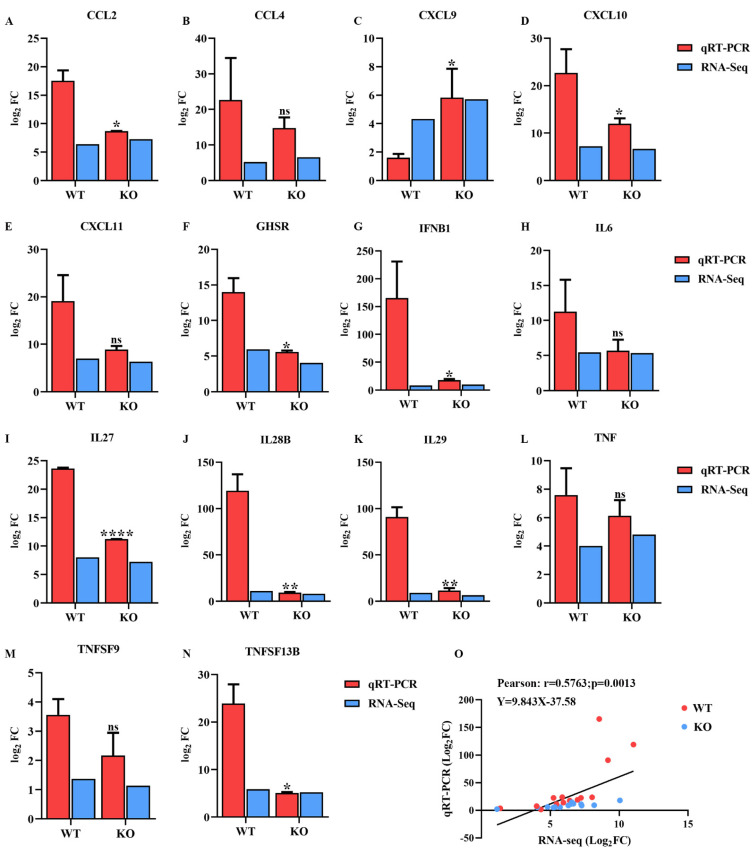
Confirmation of the transcriptome sequencing data by qRT-PCR. (**A**–**N**) DEGs related to immune and inflammatory responses were selected randomly for qRT-PCR analysis, and the expression levels of those DEGs were estimated using the 2^− ∆∆CT^ method. (**O**) The correlation of the DEG expression levels between RNA sequencing and qRT-PCR was analyzed using Pearson’s correlation analysis. * means *p* ≤ 0.05, ** means *p* ≤ 0.01, **** means *p* ≤ 0.0001.

Fourteen DEGs related to immune and inflammatory responses were randomly selected for qRT-PCR analysis to verify the expression profiles of DEGs generated using RNA sequencing. As shown in Figure 8, the expression of most of the selected DEGs generated by RNA sequencing was consistent with the levels obtained using qRT-PCR. In addition, the correlation between the expression levels of the selected DEGs obtained using RNA sequencing and qRT-PCR was analyzed by calculating Pearson’s correlation coefficient. The results revealed that Pearson’s correlation coefficient was 0.57 (*p* = 0.0013) (Figure 8O), confirming that the data generated by RNA sequencing were reliable.

### 2.8. HSP90AB1 Knockout Modulates NF-κB Activity

**Figure 9:**
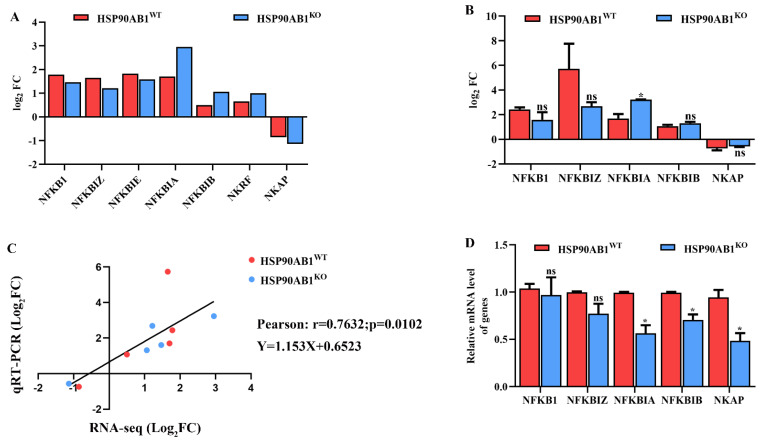
HSP90AB1 knockout alters NF-κB activity. (**A**) The expression patterns of DEGs involved in regulating NF-κB activity were analyzed using RNA sequencing. (**B**) qRT-PCR analysis of the expression of NFKB1, NFKBIZ, NFKBIA, NFKBIB and NKAP in HSP90AB1^WT^ and HSP90AB1^KO^ cells. (**C**) The correlation of the DEGs between RNA sequencing and qRT-PCR data was analyzed by calculating Pearson’s correlation coefficient. (**D**) The mRNA level of NFKB1, NFKBIZ, NFKBIA, NFKBIB and NKAP in PDCoV uninfected HSP90AB1^WT^ and HSP90AB1^KO^ cells was analyzed by qRT-PCR. * means *p* ≤ 0.05.

The expression levels of DEGs involved in regulating NF-κB activity were analyzed to determine the relationship between HSP90AB1 and the NF-κB signaling pathway. RNA sequencing results showed that the expression levels of NFKB1, NFKBIZ, NFKBIE, NFKBIA, NFKBIB and NKRF were upregulated in both cells, while the NF-κB activating protein NKAP was downregulated (Figure 9A). The mRNA level of NFKB1, NFKBIZ, NFKBIA, NFKBIB and NKAP were also analyzed using qRT-PCR. Results showed that HSP90AB1 knockout resulted in the upregulation of NFKBIA caused by PDCoV infection was enhanced, and Pearson’s correlation coefficient was 0.7632 (*p* = 0.0102) (Figure 9B,C), confirming that the RNA sequencing data were reliable and accurate. In addition, the relative mRNA level of NFKB1, NFKBIZ, NFKBIA, NFKBIB and NKAP in PDCoV uninfected HSP90AB1^WT^ and HSP90AB1^KO^ cells was further analyzed by qRT-PCR and results indicated that knockout of HSP90AB1 led to a reduction of NFKBIA, NFKBIB and NKAP mRNA level (Figure 9D). These findings indicated that HSP90AB1 knockout inhibited PDCoV replication potentially by altering NF-κB activity.

**Figure 10:**
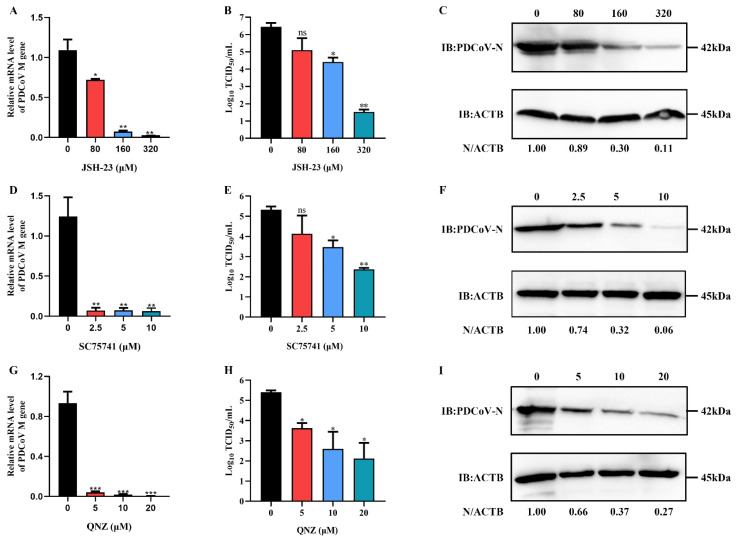
The NF-κB inhibitor reduced PDCoV infection. (**A**,**D**,**G**) qRT-PCR analysis of the mRNA level of the PDCoV *M* gene after JSH-23 (**A**), SC75741 (**D**) and QNZ (**G**) treatment. (**B**,**E**,**H**) The viral titer in the culture supernatants was determined using TCID_50_ assay. (**C**,**F**,**I**) Western blotting detection of the PDCoV N protein level after JSH-23 (**C**), SC75741 (**F**) and QNZ (**I**) treatment. ACTB was used as the sample loading control. * means *p* ≤ 0.05, ** means *p* ≤ 0.01, *** means *p* ≤ 0.001.

The NF-κB inhibitors, JSH-23, SC75741 and QNZ, were used to further evaluate whether they could inhibit PDCoV infection. The cytotoxic effect of JSH-23, SC75741 and QNZ was first determined using the CCK-8 assay. The relative cell viability decreased after treatment with increasing inhibitors concentrations, and the cell viability remained above 90% after treatment with JSH-23 at concentrations not exceeding 320 μM, and with SC75741 and QNZ at concentrations not exceeding 20 μM (Figure S4). Moreover, PDCoV infection was inhibited in LLC-PK cells treated with various concentrations of JSH-23 SC75741 and QNZ in a dose-dependent manner (Figure 10), indicating that the NF-κB signaling pathway might play a vital role in PDCoV infection.

## 3. Discussion

PDCoV is an important enteropathogen that infects mammals and avians, including swine [13], cattle [40], chicks and turkey [41,42]. To date, the pathogenesis of PDCoV is not fully understood. HSP90, an important host factor, has been reported to be involved in various cellular processes, such as cell signaling pathways, immunological functions and cell survival, and plays critical roles in the replication of several viruses [43,44,45]. In this study, we also found that HSP90AB1 knockout exerted a significant effect on PDCoV infection; however, the molecular mechanisms of HSP90AB in PDCoV infection are poorly understood. Therefore, studies aiming to explore the role and function of HSP90AB1 in PDCoV infection from the perspective of transcriptome analysis are very important because they will contribute to providing critical information for elucidating the mechanisms of interactions between pathogens and host factors.

Transcriptional and proteomic analyses have been widely used to evaluate the host cell response to virus infection [12,46,47]. Some reports have also explored the response of host cells to PDCoV infection and shown that PDCoV infection activates pattern recognition receptor (PRR)-mediated signaling pathways, such as the NOD-like receptor signaling pathway, Toll-like receptor signaling pathway, RIG-I-like receptor signaling pathway, and cytosolic DNA-sensing pathway, which mediate the production of IFN [11]. Similarly, our results indicated that the DEGs in HSP90AB1^WT^ and HSP90AB1^KO^ cells were also significantly enriched in these signaling pathways upon PDCoV infection, resulting in the upregulation of most IFNs (Figure 6 and Figure 7). Moreover, the cytokine-cytokine receptor interaction signaling pathway, TNF signaling pathway and Jak-STAT signaling pathway were also activated following PDCoV infection. Therefore, in the present study, the identified DEGs were mainly related to the immune and inflammatory responses, which modulate virus infection by regulating these cellular processes.

The MAPK and NF-κB signaling pathways are also important signaling pathways related to viral infections [48,49]. Accumulating evidence suggests that activation of the MAPK and NF-κB signaling pathways regulates proinflammatory cytokine production [50,51]. Additionally, PDCoV infection induces the secretion of the cytokines IL-6, IL-12, TNF-α, IFN-α and IFN-β via the activation of the p38/MAPK and NF-κB signaling pathways [52]. In our study, we found that most of the DEGs enriched in the NF-κB signaling pathway were upregulated upon PDCoV infection in HSP90AB1^WT^ cells (Figure 7). Previous studies reported that HSP90AB1 can interact with IKK and signaling proteins of NF-κB pathways, such as transforming growth factor beta-activated kinase 1 (TAK1) and TBK1 [37]. Moreover, the activation of NF-κB also requires HSP90 activity and HSP90 inhibitor, AT-533, impairs NF-κB activation induced by HSV-1 [53]. In the present study, the DEGs enriched in the NF-κB signaling pathway were specifically identified in HSP90AB1^WT^ cells, and the NF-κB signaling pathway identified in HSP90AB1^KO^ cells was not significantly enriched pathway upon PDCoV infection (Figure 6A,B). Further, we also found HSP90AB1 knockout resulted in the upregulation of NFKBIA caused by PDCoV infection was enhanced and led to a reduction of NFKBIA, NFKBIB and NKAP mRNA levels (Figure 9), indicating that the effect of HSP90AB1 on PDCoV infection might be related to the NF-κB signaling pathway. Previous research has also reported that the NF-κB signaling pathway might be a suitable target for antiviral intervention, and inhibition of this signaling pathway by suppressing NF-κB activity might result in a reduction in virus infection [54]. In our study, the NF-κB inhibitors, JSH-23, SC75741 and QNZ, reduced PDCoV infection at the mRNA and protein levels (Figure 10), similar to previous results obtained for some other viruses, such as avian influenza A viruses (IAVs) [55], severe fever with thrombocytopenia syndrome virus (SFTSV) and heartland virus (HRTV) [56].

Cytokines, such as IFNs, chemokines, tumor necrosis factor (TNF) and IL, play pivotal roles in the process of anti-infection immune and inflammatory responses [57]. PDCoV infection induces the release of cytokines, such as IL-6, IL-12, TNF-α, IFN-α and IFN-β, in vivo [58,59]. Moreover, the expression levels of the cytokines IFN-γ, IL-10 and TNF-α were also significantly increased in the serum and cecal tissue of PDCoV-infected chickens [60]. Coinfection with PDCoV and PEDV upregulated the expression of inflammatory cytokines, IL-6, IL-8, IL12 and TNF-α in vivo [61,62]. In the present study, the expression levels of the proinflammatory cytokines CXCL10, CXCL11, IL-6 and TNF-α were upregulated in both cell lines, especially the CXCL10 gene, which was increased by approximately 25-fold in HSP90AB1^WT^ cells (Figure 8), resulting in the development of inflammation. However, the expression level of CXCL10 in HSP90AB1^KO^ cells was lower than those in HSP90AB1^WT^ cells, indicating that HSP90AB1 knockout reduced the inflammatory response induced by PDCoV infection (Figure 8). Similar to our findings, Wyler et al. [44] found that treatment of cells with HSP90 inhibitors decreased the upregulation of proinflammatory cytokines, particularly IL-6, CXCL10 and CXCL11, during SARS-CoV-2 infection.

IFNB1, the most significantly increased IFN, was upregulated approximately 160-fold in HSP90AB1^WT^ cells (Figure 8), exerting a vital antiviral function in the host innate immune response. Additionally, PDCoV infection increased the transcription of the chemokines CCL2, CCL4, CXCL9, CXCL10 and CXCL11 (Figure 8), suggesting that these chemokines may contribute to the recruitment and regulation of macrophages or other inflammatory cells to the sites of virus infection [63]. Nevertheless, the upregulation of the abovementioned cytokines, CXCL10 and CCL2, was reduced, in HSP90AB1^KO^ cells, while the upregulation of CXCL9 was enhanced (Figure 8). The cytokines IL28B and IL29, which are members of the type III IFN family, were significantly upregulated in HSP90AB1^WT^ cells compared with HSP90AB1^KO^ cells (Figure 7 and Figure 8). We suggested that this phenomenon might be related to the activation of the MAPK signaling pathway in HSP90AB1^WT^ cells, which regulates type III IFN gene expression [64].

To our knowledge, this report is the first to investigate the role of HSP90AB1 in PDCoV infection by performing a comparative transcriptome analysis. We compared the transcriptome alterations in HSP90AB1^WT^ and HSP90AB1^KO^ cells upon PDCoV infection. A total of 1295 and 3746 DEGs were identified in PDCoV-infected HSP90AB1^WT^ and HSP90AB1^KO^ cells, respectively. GO enrichment analysis revealed that a majority of the DEGs were enriched in cellular processes of BP, and the most annotated CC and MF terms were cell and cell parts, and binding and protein binding, respectively. KEGG pathway enrichment analysis indicated that the most significantly enriched pathways were related to immune and inflammatory responses. Moreover, DEGs enriched in the NF-κB signaling pathways were specifically detected in HSP90AB1^WT^ cells. Further validation of immune and inflammatory response-related genes suggested that most cytokines were upregulated upon PDCoV infection and knockout of HSP90AB1 altered the expression levels of some cytokines associated with immune and inflammatory responses. In conclusion, comparative transcriptomic analysis revealed that HSP90AB1 was related to the immune and inflammatory responses to PDCoV infection.

## 4. Materials and Methods

### 4.1. Cells, Virus, Inhibitors and Antibodies

LLC-PK and HEK293T cells were maintained in Dulbecco^’^s modified Eagle’s medium (DMEM) (Gibco, USA) supplemented with 10% fetal bovine serum (FBS) (PNA, Germany) and a 1% antibiotic-antimycotic solution (Solarbio, China) at 37 °C in a humidified atmosphere of 5% CO_2_. PDCoV strain CHN-SC2015 (GenBank accession no. MK355396) was isolated in Sichuan Province in 2018 [1]. The NF-κB inhibitor JSH-23 (CAS No. 749886-87-1), SC75741 (CAS No. 913822-46-5) and QNZ (CAS No. 545380-34-5) were purchased from Selleck Chemicals (Houston, TX, USA). The anti-PDCoV N mouse monoclonal antibody was prepared by our laboratory [65]. The anti-HSP90AB1 rabbit polyclonal antibody (11405-1-AP) was purchased from Proteintech Group (Chicago, IL, USA). HRP-conjugated goat anti-mouse IgG (AS003), HRP-conjugated goat anti-rabbit IgG (AS014), the anti-HSP70 rabbit polyclonal antibody (A12948) and the anti-ACTB rabbit polyclonal antibody (AC026) were purchased from Abclonal (Wuhan, China).

### 4.2. Generation of HSP90AB1 Knockout Cells Using the CRISPR/Cas9 System

A single-guide RNA (sgRNA) targeting exon 7 was synthesized and inserted into the vector lentiCRISPR v2 (Addgene plasmid 52961) to generate a deletion or insertion mutation within the *HSP90AB1* gene in LLC-PK cells. The sgRNA sequences were as follows: sgRNA-F, CACCGAGGTCAAAAGGAGCCCGACG; and sgRNA-R, AAACCGTCGGGCTCCTTTTGACCTC. The sgRNA expression plasmid was packaged into lentivirus particles in HEK293T cells by cotransfection with psPAX2 (Addgene plasmid 12260) and pMD2.G (Addgene plasmid 12259) at a ratio of 5:3:2. Forty-eight hours after transfection, supernatants were collected and centrifuged for 10 min at 5000 rpm at 4 °C. The lentiviruses were then transduced into LLC-PK cells that were approximately 50% confluent. After infection for 36 h, cells were subjected to selection with 4 μg/mL puromycin (Solarbio, China) for seven days. Genomic DNA was extracted from puromycin-resistant LLC-PK cells using the TIANamp Genomic DNA Kit (TianGen, Beijing, China). The *HSP90AB1* gene was detected in the purified genomic DNA by PCR amplification with primers that annealed approximately 250 bp upstream and downstream from the target site. The primer sequences were as follows: F, TGGATTTCTCTCACAGCACTTC; R, TTCCTTTGAGAAAGCTGAGACC. The PCR products were sequenced by Sangon Biotech (Shanghai, China). HSP90AB1 knockout clonal cell lines (HSP90AB1^KO^) were isolated by dilution and identified using qRT-PCR and western blotting. Subsequently, HSP90AB1 wild-type cells (HSP90AB1^WT^) and HSP90AB1^KO^ cells were infected with PDCoV at a multiplicity of infection (MOI) of 0.1. Supernatants and cell suspensions were harvested at 24 h postinfection and analyzed by measuring the TCID_50_ and qRT-PCR, respectively. The remaining cells were lysed and analyzed using western blotting.

### 4.3. Sample Preparation and RNA Extraction

HSP90AB1^WT^ and HSP90AB1^KO^ cells seeded in T75 cell culture flasks were infected with PDCoV at an MOI of 0.1. Meanwhile, the mock-infected cells served as a control. Two flasks of cells from each group were used as two independent biological replicates for a total of eight samples. Twenty-four hours postinfection, cell samples were collected and lysed using TRIzol reagent (Beyotime, Shanghai, China), and total RNA was extracted using a TRIzol reagent kit (Invitrogen, Carlsbad, CA, USA) according to the manufacturer’s protocol. RNA quality was assessed using an Agilent 2100 Bioanalyzer (Agilent Technologies, Palo Alto, CA, USA) and RNase-free agarose gel electrophoresis. RNA samples with RNA integrity numbers (RINs) greater than 8 were selected for subsequent library preparation and sequencing.

### 4.4. cDNA Library Preparation and Sequencing

The mRNA was first enriched from the total RNA using oligo (dT) beads. Then, the enriched mRNA was segregated into short fragments using fragmentation buffer and reverse transcribed into cDNAs with random hexamer primers and M-MuLV reverse transcriptase. Second-strand cDNAs were synthesized by DNA polymerase I, RNase H, dNTPs and buffer with first-strand cDNAs as templates. Then, the cDNA fragments were purified with a QiaQuick PCR extraction kit (Qiagen, Venlo, The Netherlands), end repaired, subjected to A base addition, and ligated to Illumina sequencing adapters. The ligation products were selected by size through agarose gel electrophoresis, amplified by PCR, and sequenced using an Illumina NovaSeq6000 platform by Gene Denovo Biotechnology Co. (Guangzhou, China).

### 4.5. Read Quality Control and Mapping

High-quality clean reads were further filtered from raw reads by removing reads containing sequencing adapters or more than 10% of unknown nucleotides (N), as well as low-quality reads with more than 50% of low-quality (Q-value ≤ 20) bases using fastp (version 0.18.0). At the same time, the parameters Q20, Q30 and guanine-cytosine (GC) content of clean data were also calculated. Residual ribosomal RNA (rRNA) was removed by mapping the clean reads to the rRNA database using the short read alignment tool Bowtie2 (version 2.2.8). An index of the *Sus scrofa* 11.1 reference genome (https://ftp.ensembl.org/pub/release-100/fasta/sus_scrofa/dna/ (accessed on 28 February 2021)) was built and paired-end clean reads were mapped to the reference genome using HISAT2.2.4 with “-rna-strandness RF” and default settings of the other parameters. The mapped reads of each sample were assembled using StringTie v1.3.1 software, and an FPKM (fragment per kilobase of transcript per million mapped reads) value for each transcript region was calculated to quantify its expression abundance and variations using RSEM software.

### 4.6. Correlation Analysis of Samples

The correlation analysis was performed using R. Correlations between two parallel experiments were determined to evaluate the reliability of experimental results and operational stability. Pearson’s correlation coefficients for the correlations within and between groups were calculated, and a heatmap was drawn. Principal component analysis (PCA) was then performed with the R package gmodels (http://www.r-project.org/ (accessed on 28 February 2021)) to evaluate the differences between groups and the repetition of samples within groups.

### 4.7. Differentially Expressed Gene (DEG) Analysis

The DEG analysis was performed between mock- and PDCoV-infected cells using DESeq2 software under the condition of a false discovery rate (FDR) ≤ 0.05 and |log_2_ fold change| ≥ 1. Subsequently, all DEGs were mapped to Gene Ontology (GO) terms in the GO database (http://www.geneontology.org/(accessed on 28 February 2021)), and gene numbers were calculated for every term. Significantly enriched GO terms (FDR-corrected *p*-value ≤ 0.05) in DEGs compared to the genome background were defined by hypergeometric testing. Pathway enrichment analysis was also performed using the Kyoto Encyclopedia of Genes and Genomes (KEGG) database. Pathways with FDR-corrected *p* values ≤ 0.05 were defined as significantly enriched pathways in DEGs.

### 4.8. qRT-PCR

Total RNA was extracted from PDCoV-infected cells using the UNIQ-10 column TRIzol Total RNA Isolation Kit (Sangon, China) and subjected to reverse transcription using the PrimeScript RT Reagent Kit with gDNA Eraser (Takara, China). The cDNA templates were used for qRT-PCR with TB Green Premix Ex Taq II (Takara, China) and the specific primers shown in Table 3. Relative mRNA levels of the gene were calculated using the 2^-ΔΔCT^ method with the porcine *ACTB* gene serving as an internal control. The qRT-PCR conditions were 95 °C for 30 s, followed 40 cycles of 95 °C for 5 s, 55 °C for 30 s, and 72 °C for 30 s using the LightCycler 96 system (Roche, Germany). Each experiment consisted of three biological replicates, and qRT-PCR for each sample was performed in triplicate.

### 4.9. Western Blotting

HSP90AB1^WT^ and HSP90AB1^KO^ cells were collected at 24 h postinfection, washed twice with cold PBS, and then incubated with RIPA lysis buffer containing the protease inhibitor PMSF (Solarbio, Beijing, China) for 15 min at 4 °C. Lysates were centrifuged at 12,000 rpm for 5 min at 4 °C, and the supernatants were collected. Ten microliters of supernatant were subjected to SDS–PAGE under reducing conditions and transferred to PVDF membranes (Bio-Rad, Hercules, CA, USA) for 1 h at 250 mA. Membranes were blocked with 5% (*w*/*v*) skim milk in TBST (0.05% Tween 20, 0.15 M NaCl, and 1 M Tris-HCI, pH 7.5) for 90 min at room temperature and then incubated overnight with primary antibodies diluted in primary antibody dilution buffer (Beyotime, China). An anti-ACTB rabbit polyclonal antibody was used a loading control to confirm equal protein sample loading. Membranes were washed four times for 5 min each with TBST and then incubated for 45 min at room temperature with a 1:5000 dilution of HRP-conjugated goat anti-rabbit or anti-mouse IgG. Membranes were washed again and developed by adding ECL reagents (Bio-Rad, Hercules, CA, USA) according to the manufacturer^’^s instructions. The band density of each protein was quantified using ImageJ software.

### 4.10. TCID_50_

The confluent cell monolayers seeded in 96-well plates were washed twice with DMEM supplemented with 5 μg/mL trypsin (referred to as maintenance medium) and then inoculated with 100 μL of 10-fold serial dilutions of PDCoV; eight technical replicates of each dilution were established. Then, 150 μL of maintenance medium were added to each well after the cells and virus had incubated for 1.5 h at 37 °C. The CPE was observed for 4 days and was analyzed using the methods described by Reed & Muench [66].

### 4.11. Cell Viability Assay

The cytotoxicity of the NF-κB inhibitor JSH-23, QNZ and SC75741 was assessed using the Cell Counting Kit-8 (CCK-8) (Meilun, Dalian, China). Briefly, 100 µL of 10^5^ LLC-PK cells/mL were aliquoted into wells of 96-well plates and then returned to the incubator for 24 h. Next, the cells were treated with a series of JSH-23 concentrations in serum-free DMEM for 24 h. Mock-treated cells and DMEM without inhibitor served as the negative control and blank control, respectively. After washes with PBS, 10 μL of CCK-8 reagent were added to each well, and the cells were incubated for 1 h at 37 °C. Absorbance was measured at a wavelength of 450 nm using a microplate reader (Thermo Scientific, Waltham, MA, USA). Cytotoxicity was calculated as [(mean OD_450_ inhibitor-mean OD_450_ blank)/(mean OD_450_ control-mean OD_450_ blank)] × 100%.

### 4.12. Statistical Analysis

The methods used for the statistical analysis of transcriptome sequencing data are described in Section 4.7. Data from three independent qRT-PCR assays are presented as the means ± standard deviations (SD). The student’s *t*-test was used to measure significant differences between two groups with GraphPad Prism software. *p* values < 0.05 were considered statistically significant.

## Figures and Tables

**Table 1:** Summary statistics for quality control of the sequencing data.

Sample	Raw Reads	Clean Reads	Raw Bases (G)	Clean Bases (G)	Q20 (%)	Q30 (%)	GC Content (%)
KO-PDCoV-1	89,417,288	89,203,130	13.41	13.28	97.68	93.27	48.14
KO-PDCoV-2	73,552,754	73,402,918	11.03	10.93	97.73	93.40	48.58
KO-mock-1	69,435,150	69,244,218	10.42	10.32	97.57	93.06	48.68
KO-mock-2	60,095,972	59,938,292	9.01	8.93	97.61	93.14	48.79
WT-PDCoV-1	68,873,684	68,723,094	10.33	10.24	97.55	92.88	48.10
WT-PDCoV-2	72,214,636	72,048,968	10.83	10.74	97.60	93.06	48.03
WT-mock-1	84,349,756	84,131,302	12.65	12.53	97.52	92.90	48.49
WT-mock-2	67,038,012	66,866,762	10.06	9.95	97.60	93.05	48.88

**Table 2:** Summary statistics of clean reads mapped to the reference genome.

Sample	Total Reads ^1^	Total Mapped Reads (%)	Unique Mapped Reads (%)	Exon (%)	Intron (%)	Intergenic (%)	Expressed Genes	Novel Genes
KO-PDCoV-1	89,079,788	85.68	83.21	76.03	20.96	3.01	15,299	581
KO-PDCoV-2	73,291,444	85.19	82.75	77.71	19.47	2.82	15,073	588
KO-mock-1	69,123,276	94.99	92.47	78.22	18.27	3.51	15,260	592
KO-mock-2	59,835,550	95.11	92.63	78.09	18.43	3.48	15,246	587
WT-PDCoV-1	68,625,598	86.24	83.86	79.22	17.75	3.04	15,634	589
WT-PDCoV-2	71,947,570	87.61	85.25	79.09	17.82	3.09	15,745	587
WT-mock-1	84,004,888	95.48	92.86	79.09	17.70	3.21	15,786	586
WT-mock-2	66,758,268	95.30	92.64	79.72	17.16	3.12	15,485	579

^1^ Total reads: clean reads were filtered from the ribosomal RNA-mapped reads.

**Table 3:** The primers used for qRT-PCR in this study.

Genes	Sequences (5′–3′)	Size (bp)
PDCoV M	F: CCAATGGGTACATGGAGGT	130
	R: GTGGCGGATTTCTAACTGA	
HSP90AB1	F: TGGAGAGTTCTACAAAAGCCTG	304
	R: CTTCAAAATCTTGCTCTGCTG	
HSP70	F: GCACGAGGAAAGCCTTAGAG	166
	R: GGAGAAGATGGGACGACAAA	
CCL4	F: CTCTCCTCCAGCAAGACCAT	296
	R: CAGTTCAGTTCCAAGTCATCCA	
CXCL10	F: AATCTACCTCTGCCATCATCTC	373
	R: AGTAGAAGCCCACGGAGTAAAG	
CXCL11	F: AACTATTCAAGGCTTCCCCAT	201
	R: ACATTTGCTTGCTTTGATTTG	
GHSR	F: TCCCACCGATGACGAGAG	276
	R: CTTGATGATTCCCAGGAGTTT	
CCL2	F: GAAGAGTCACCAGCAGCAAG	144
	R: AAGGCTTCGGAGTTTGGTT	
CXCL9	F: GCTTTTGGGTATCATCTTCCT	266
	R: TCTTTAGGCTGACCTGTTTCT	
IFNB1	F: ACCAACAAAGGAGCAGCAA	266
	R: ATCCATCTGCCCATCAAGTT	
IL27	F: CCAGATGGCAGACGACCT	185
	R: GGACCCGAACCTCAGACAG	
IL28B	F: GTGCTGATGACGGTGGCT	164
	R: AGGGACTCTTCAAAGGCATC	
IL29	F: CTTTCAAGCCCACCACAAC	241
	R: GCCCTTAGGACCTTCAGAGT	
IL6	F: GATGCTTCCAATCTGGGTTC	218
	R: ATTTGTGGTGGGGTTAGGG	
TNF	F: TCCTCACTCACACCATCAGC	222
	R: GCCCAGATTCAGCAAAGTCC	
TNFSF9	F: GGCTTCCCAGACTATCCAC	124
	R: CACCTTTCATCCCTGGCTC	
TNFSF13B	F: CCGATAACACCTTTGCCAT	124
	R: TTATTGGGTAGTGTTTCAGGC	
NFKB1	F: CGGTGAAGGTCTGAACGC	188
	R: GGGCATCACCCTCCAAGA	
NFKBIA	F: CACCAACCAGCCAGAAATC	261
	R: GCACCCAAAGACACCAACA	
NFKBIZ	F: CAAGTAGAGCAGGAAGAAAGCA	195
	R: CTGAAAGGCACTCTGTCCATTA	
NFKBIB	F: CTCTGACCATACCCCTGACA	176
	R: GGACCATCTCGGCATCTT	
NKAP	F: AGAGTCCCAGGAAGAGTTGC	153
	R: CCTTCACCAGGTAACAGAGC	
Porcine ACTB	F: CTTCCTGGGCATGGAGTCC	201
	R: GGCGCGATGATCTTGATCTTC	

## Data Availability

Not applicable.

## Supplementary Materials

The following are available online at https://www.mdpi.com/article/10.3390/ijms23063280/s1.
