# Supplementary material for: A Comparative Transcriptomic Analysis Reveals That HSP90AB1 Is Involved in the Immune and Inflammatory Responses to Porcine Deltacoronavirus Infection
Source: Int J Mol Sci. 2022 Mar 18;23(6):3280. doi: 10.3390/ijms23063280 (PMC8953809; doi:10.3390/ijms23063280)
Supplement: Supplementary file 1 [file ijms-23-03280-s001.zip › Figure S2 GO enrichment analysis of the DEGs identified in HSP90AB1WT (A) and HSP90AB1KO (B) cells following PDCoV infection.pdf]

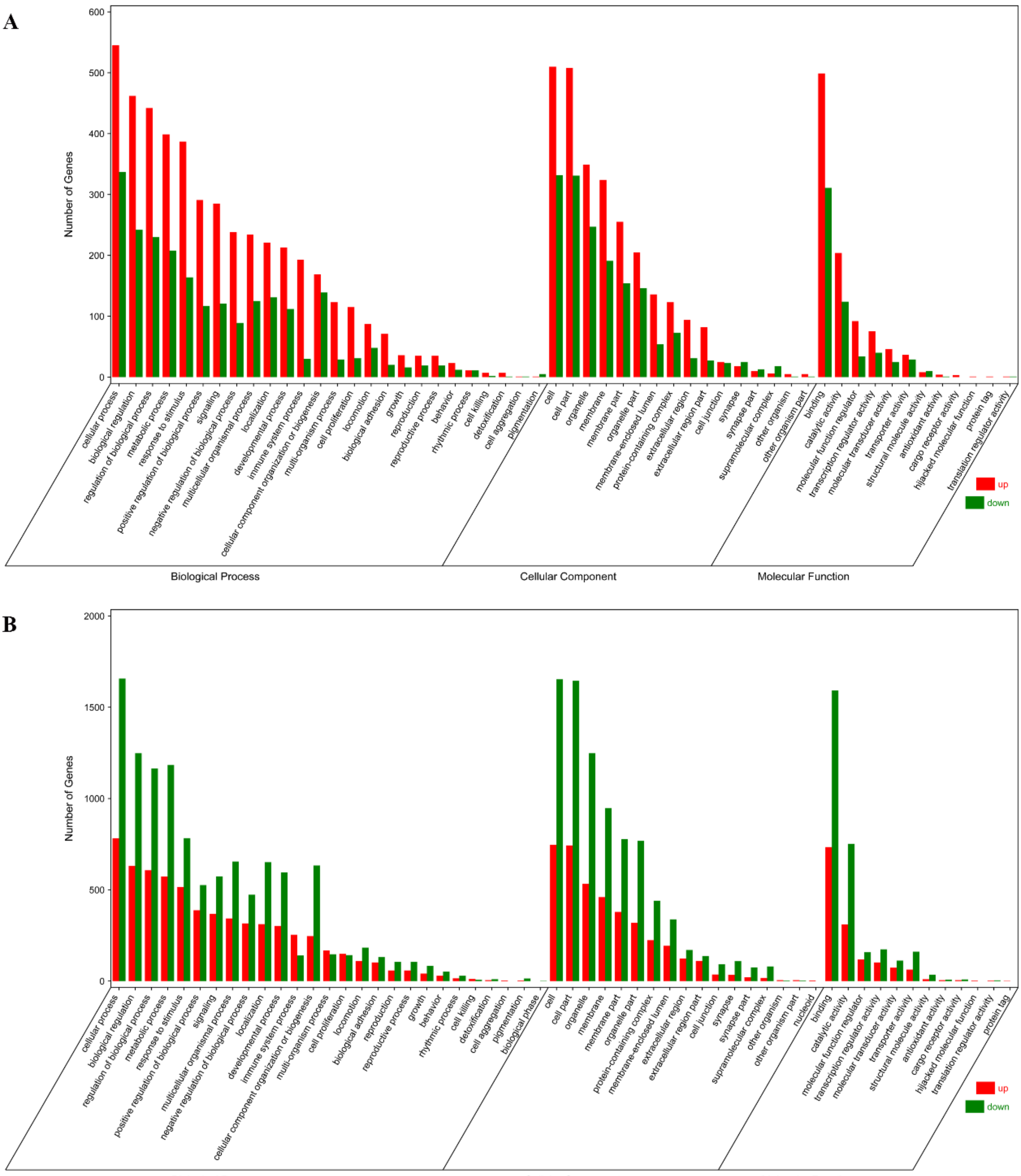

Figure S2 GO enrichment analysis of the DEGs identified in HSP90AB1^{WT} (A) and HSP90AB1^{KO} (B) cells following PDCoV infection.
