# Supplementary material for: A Comparative Transcriptomic Analysis Reveals That HSP90AB1 Is Involved in the Immune and Inflammatory Responses to Porcine Deltacoronavirus Infection
Source: Int J Mol Sci. 2022 Mar 18;23(6):3280. doi: 10.3390/ijms23063280 (PMC8953809; doi:10.3390/ijms23063280)
Supplement: Supplementary file 1 [file ijms-23-03280-s001.zip › Figure S4 The cytotoxicity of NF-a╩B inhibitors toward LLC-PK cells..pdf]

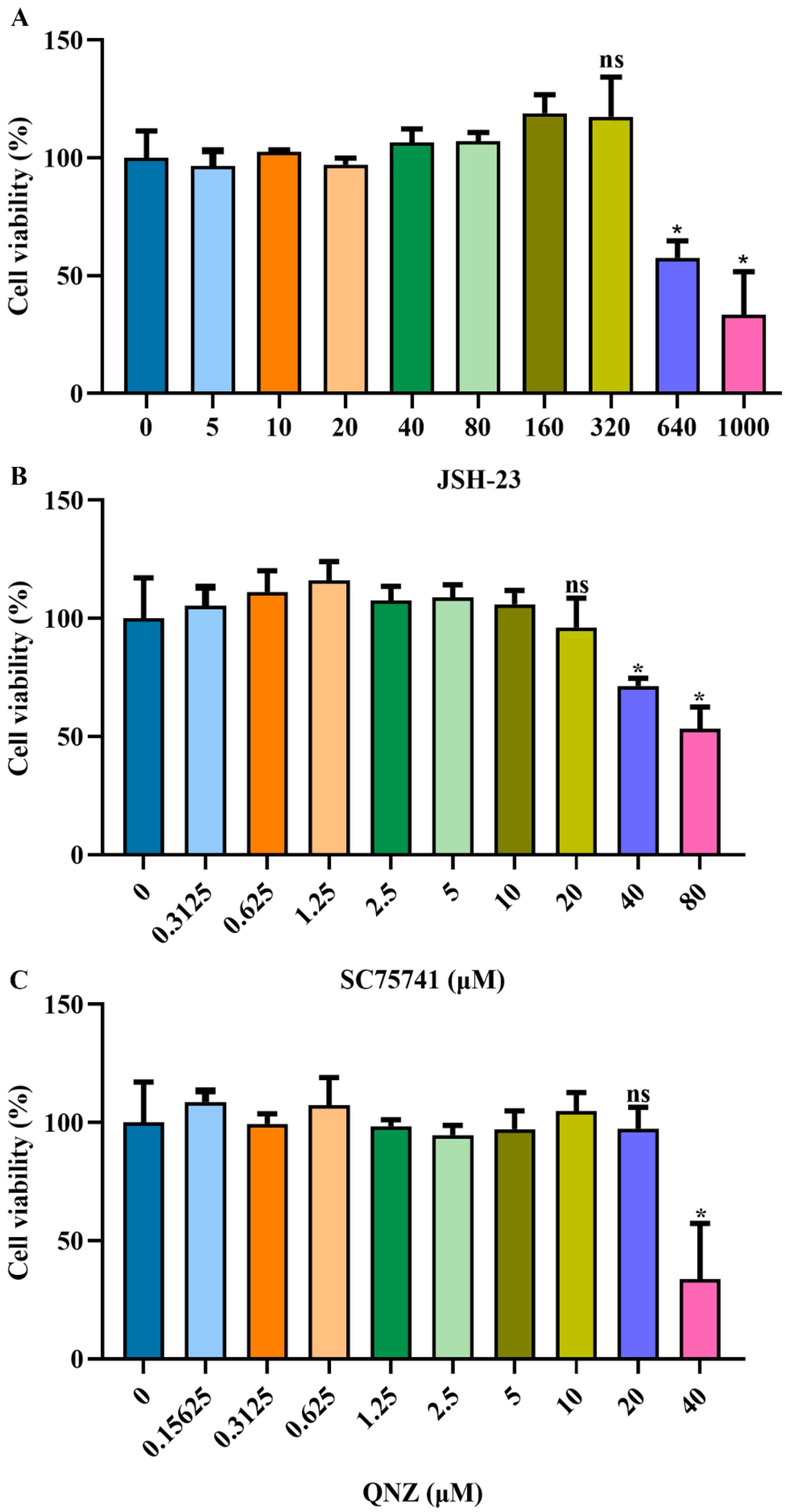

Figure S4. The cytotoxicity of NF-κB inhibitors toward LLC-PK cells. Cell viability was analyzed at 24 h posttreatment by JSH-23 **(A)** SC75741 **(B)** and QNZ **(C)** using the CCK-8 assay.
