# Supplementary figures and images for: A Comparative Transcriptomic Analysis Reveals That HSP90AB1 Is Involved in the Immune and Inflammatory Responses to Porcine Deltacoronavirus Infection
Source: Int J Mol Sci. 2022 Mar 18;23(6):3280. doi: 10.3390/ijms23063280 (PMC8953809; doi:10.3390/ijms23063280)

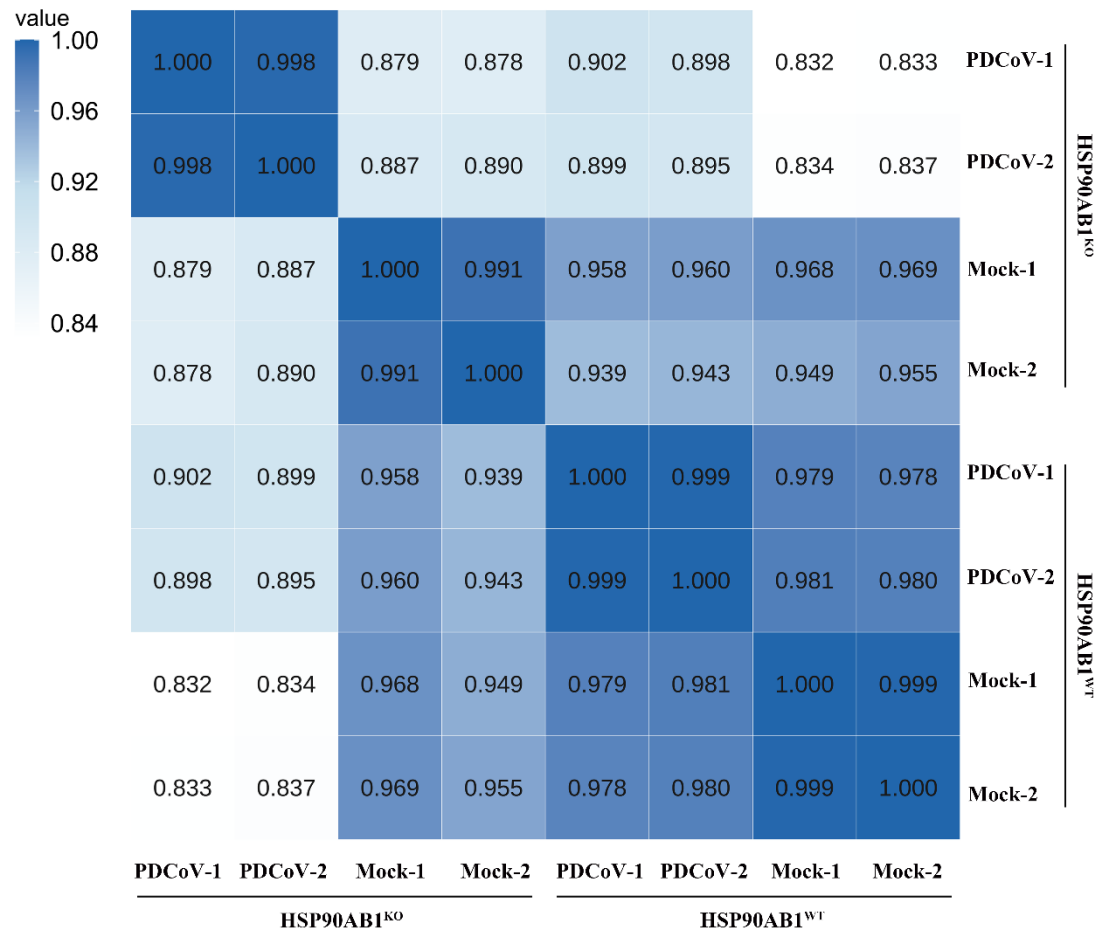

**Figure S1. Heatmap showing the correlations between biological replicates.**

Supplement: Supplementary file 1 [file ijms-23-03280-s001.zip › Figure S1 Heatmap showing the correlations between biological replicates..pdf]
